# Blood Utilization Review Using Transfusion Indices in a Tertiary Care Center: A Retrospective Observational Study

**DOI:** 10.7759/cureus.88344

**Published:** 2025-07-20

**Authors:** Saurabh Lahare, Rakesh Kumar, Bankim Das, Neha Singh

**Affiliations:** 1 Transfusion Medicine and Blood Bank, All India Institute of Medical Sciences, Patna, Patna, IND

**Keywords:** blood utilization review, ct ratio, department-wise blood utilization, msbos, transfusion index, transfusion indices, transfusion probability

## Abstract

Background

Excessive preoperative blood requisition often results in non-utilization, aging, and wastage of blood units and resources. Transfusion indices help assess the efficiency of the blood requisition process. Hence, this study aimed to analyze blood utilization patterns using transfusion indices across various departments in a tertiary care center.

Methodology

This study employed a retrospective, observational design, utilizing blood bank records collected over a one-year period from April 1, 2023, to March 31, 2024. A total of 5,881 patients were analyzed. The primary aim was to evaluate blood utilization efficiency by calculating key transfusion indices based on the crossmatching and transfusion of packed red blood cells (PRBCs). These indices included the crossmatch-to-transfusion (C/T) ratio, transfusion probability (TP), and transfusion index (TI).

Results

Out of 6,694 blood requisitions received during the study period, 5,881 patients underwent PRBC transfusions. A total of 11,894 PRBC units were crossmatched, of which 7,759 (65.23%) units were ultimately transfused. Transfusion indices demonstrated notable variations across different departments. The General Surgery Department recorded the highest C/T ratio at 2.05, while Hemato-oncology and Radiotherapy achieved the highest TP of 96%. In contrast, Dermatology reported the lowest TP at 74%. Gastro-surgery stood out with the highest TI of 2.47. Despite these differences, all departments operated within the established acceptable transfusion thresholds, maintaining C/T ratios below 2.5, TP values above 30%, and TI levels exceeding 0.5.

Conclusions

Departments with outlier indices should adopt restrictive transfusion practices. Educational programs and department-specific guidelines can optimize blood utilization, reducing unnecessary transfusions, costs, and resource consumption.

## Introduction

According to the World Health Organization’s 2016 Global Status Report on Blood Safety and Availability, the annual whole blood donation rate was 32.1 donations per 1,000 people in high-income countries, compared to just 4.6 in low-income nations. Despite comprising only about 19% of the global population, high-income countries accounted for over half of all donated blood [1]. Physicians often request more blood units than are ultimately used to maintain a safety buffer in case of unexpected bleeding. Additionally, preoperative blood orders are often influenced by existing transfusion habits rather than the actual guidelines and need [2]. This tendency to over-order, particularly before surgeries, is widespread and leads to inefficiencies such as stockpiling in blood, aging and expiry of blood units, and waste of both consumables and staff time. Excessive requests with low actual usage contribute significantly to the wastage of blood, reagents, and human resources [3,4]. Healthcare expenses, including blood usage and cost, are increasing day by day due to the inappropriate usage of medical technology and blood, leading to additional costs in the treatment of disease [5]. We aimed to evaluate the blood-ordering and transfusion practices at a tertiary care hospital using transfusion indices.

A study conducted by Mammen et al. (2022) in India reported that the clinical demand for India was estimated at 14·6 million whole blood units, which is equivalent to 36.3 donations per 1,000 eligible populations. The medicine specialty accounted for 6.0 million units, followed by surgery at 4.1 million, obstetrics and gynecology at 3.3 million, and pediatrics at 1.2 million units. The supply was 93% which was equivalent to 33.8 donations and indicated a demand and supply gap of 2.5 donations per 1,000 eligible persons, which is around one million units, illustrating a disproportion between supply and demand of blood units [4]. Safe and affordable surgeries when needed depend on a sufficient and safe blood supply, which is a common problem in our country [6]. Maximum Surgical Blood Ordering Schedule (MSBOS) protocols can be developed for the number of blood units to be crossmatched before different types of surgeries, so that work hours could be appropriately managed (e.g., following type and screen policy [7]) in the blood bank, and the required number of blood units will be ready before different types of surgeries. Clinicians often request more blood units than are ultimately used [2], prompting the development of various evaluation tools, such as transfusion indices (crossmatch-to-transfusion (C/T) ratio, transfusion probability (TP), and transfusion index (TI)), to measure the efficiency of blood ordering relative to actual usage. In the 1970s, Boral and Henry introduced the C/T ratio, considering a ratio of 2.5:1 or lower to reflect appropriate blood use, with an ideal ratio being 1.0, meaning all crossmatched blood is transfused [6]. Later, in 1980, Mead et al. introduced the TP, calculated as the number of patients transfused divided by the number of patients crossmatched, multiplied by 100. A TP of 30% or higher was deemed indicative of proper blood utilization [8]. Additionally, the TI, representing the average number of units transfused per patient crossmatched, was introduced. A TI of 0.5 or above signifies efficient and appropriate blood usage [9,10]. A study to evaluate blood transfusion patterns has not been conducted previously in the state of Bihar, India.

## Materials and methods

Study area

This study was conducted at the Department of Transfusion Medicine and Blood Bank, All India Institute of medical Sciences, Patna, Bihar, where all the specialty and superspecialty services are provided. The hospital is located in Phulwarisharif area of Patna, Bihar.

Study design

In this observational, retrospective study, data were collected from blood bank registries. Patient requisition forms, crossmatch register, and issue register were searched for one year (April 01, 2023, to March 31, 2024), resulting in a total of 5,881 patients. Data from all patients for whom blood requisition forms were sent mentioning packed red blood cell (PRBC) crossmatching were included in this study. Data for all these patients were collected from the blood bank registries to calculate the following transfusion indices [6,11-15]: C/T ratio = number of units crossmatched/number of units transfused; TP = number of patients transfused/number of patients crossmatched × 100; TI = number of units transfused/number of patients crossmatched.

This study aimed to investigate the pattern of blood utilization using transfusion indices among patients admitted to a tertiary care health center. By evaluating transfusion indices across different departments, the study sought to assess the efficiency of blood requisition and utilization practices. The primary objective is to analyze department-wise transfusion indices to identify trends and optimize blood resource management. The study encompasses all patients who requested crossmatching during the one-year period from April 1, 2023, to March 31, 2024, analyzing a total of 5,881 patients. This comprehensive approach ensures a thorough evaluation of blood requisition patterns and their impact on transfusion rates across various medical and surgical specialties.

**Figure 1:**
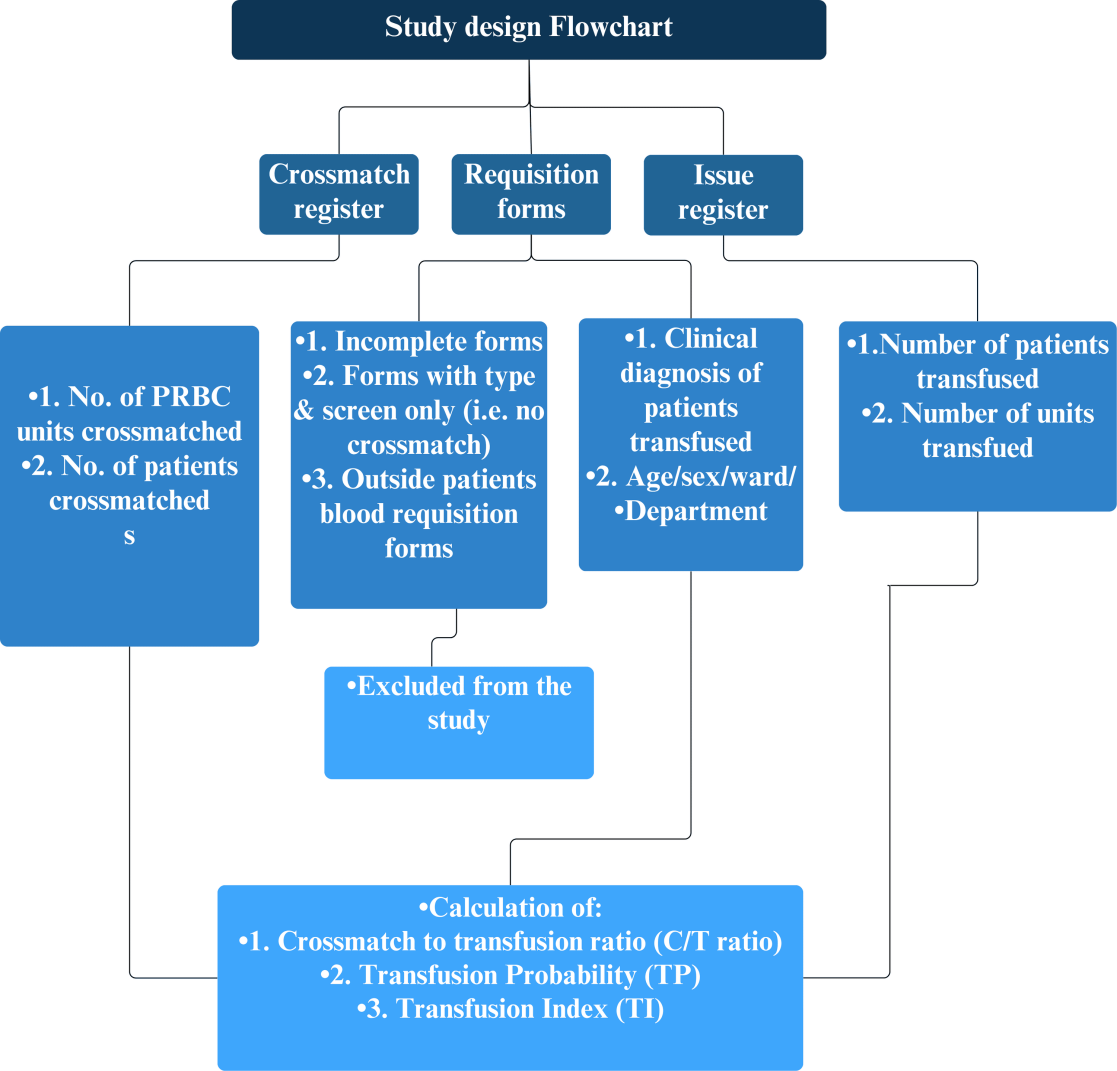
Study design flowchart.

The study included all patients within the specified period who had a PRBC crossmatching request. Additionally, scheduled surgical patients whose procedures were canceled after PRBC crossmatching and cases where PRBC units were returned after issuance were also considered for analysis. However, certain requisitions were excluded from the study. Incomplete forms were omitted to ensure data accuracy and reliability. Blood requisitions that involved only type and screen requests, without crossmatching, were also excluded. Furthermore, blood requisition forms from outside patients were not considered part of the study population. Figure 1 presents the study flowchart.

## Results

**Figure 2:**
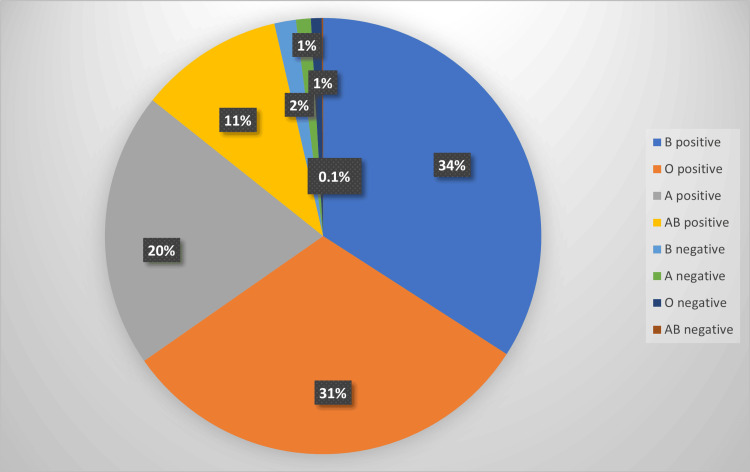
Blood group-wise transfusion data.

A total of 6,694 requisitions came for PRBC crossmatching from different surgical and non-surgical departments. In total, 5,881 patients were transfused with PRBC units, 11,894 PRBC units were crossmatched, and 7,759 (65.23%) PRBC units were transfused. Overall C/T ratio was 1.53, TP was 88%, and TI was 1.15. The Department of General Medicine had a maximum patient requisitions of 1,337 (19.9%) for PRBC crossmatching, with 1,233 (15.9%) PRBC units transfused. The Department of Ophthalmology had a minimum number of patient requisitions of 16 (0.23%), with 16 (0.18%) PRBC units transfused. The Department of General Surgery had the maximum C/T ratio of 2.05, the Department of Hemato-oncology, CTVS, and Radiotherapy had the maximum TP of 96%, and the Department of Dermatology had the minimum TP value of 74%. The Department of Gastro-surgery had the maximum TI of 2.47. Blood group-wise transfusion data are presented in Figure 2, with B-positive having 34% transfusions and AB-negative having 0.1% transfusions. All the departments had their transfusion indices within the normal range (i.e., C/T ratio <2.5, TP >30%, TI >0.5). Department-wise blood utilization data and transfusion indices data are presented in Table 1 and Table 2, respectively.

**Table 1:** Department-wise blood utilization data. PRBCs: packed red blood cells

Department	Number of patients crossmatched	Number of patients transfused	Number of PRBCs crossmatched	Number of PRBCs transfused
Pediatrics	798 (11.92%)	644 (10.95%)	826 (6.94%)	686 (5.76%)
General Surgery	434 (6.48%)	392 (6.66%)	1,665 (13.99%)	812 (6.82%)
Gastro-surgery	238 (3.55%)	224 (3.80%)	644 (5.41%)	588 (4.94%)
Oncosurgery	196 (2.92%)	182 (3.09%)	350 (2.94%)	308 (2.58%)
Orthopedics	406 (6.06%)	364 (6.18%)	697 (5.86%)	423 (3.55%)
Obstetrics & Gynecology	891 (13.31%)	717 (12.19%)	1,714 (14.41%)	843 (7.08%)
Trauma & Emergency	537 (8.02%)	464 (7.88%)	1,383 (11.62%)	761 (6.39%)
Gastro-surgery	241 (3.60%)	226 (3.84%)	463 (3.89%)	339 (2.85%)
Urology	173 (2.58%)	153 (2.60%)	227 (1.90%)	169 (1.42%)
CTVS	178 (2.65%)	171 (2.90%)	353 (2.97%)	253 (2.12%)
Neurosurgery	183 (2.73%)	173 (2.94%)	337 (2.83%)	241 (2.02%)
Plastic Surgery	31 (0.46%)	29 (0.49%)	97 (0.81%)	71 (0.59%)
Pediatric Surgery	129 (1.92%)	123 (2.09%)	153 (1.28%)	126 (1.05%)
General Medicine	1,337 (16.98%)	1,163 (19.77%)	1,737 (14.60%)	1,233 (10.3%)
Hemato-oncology	334 (4.98%)	319 (5.42%)	419 (3.52%)	331 (2.78%)
Radiotherapy	226 (3.37%)	217 (3.68%)	326 (2.74%)	223 (1.87%)
Emergency Medicine	213 (3.18%)	198 (3.36%)	317 (2.66%)	222 (1.86%)
Pulmonary Medicine	29 (0.43%)	27 (0.45%)	43 (0.36%)	29 (0.24%)
Dermatology	31 (0.46%)	23 (0.39%)	41 (0.34%)	24 (0.2%)
Ophthalmology	16 (0.23%)	13 (0.22%)	19 (0.15%)	14 (0.11%)
ENT	73 (1.09%)	59 (1.00%)	97 (0.81%)	63 (0.52%)
Total	6,694	5,881	11,894	7,759

**Table 2:** Department-wise transfusion indices data.

Departments	Crossmatch to transfusion ratio	Transfusion probability	Transfusion index
Paediatrics	1.2	81%	0.86
General Surgery	2.05	90%	1.87
Gastro Surgery	1.09	94%	2.47
Onco-surgery	1.13	93%	1.57
Orthopedics	1.64	89%	1.04
Obstetrics & Gynecology	2.03	91%	0.95
Trauma & Emergency	1.81	80%	1.42
Gastro-surgery	1.36	94%	1.41
Urology	1.34	88%	0.98
CTVS	1.39	96%	1.42
Neurosurgery	1.4	95%	1.32
Plastic Surgery	1.37	94%	2.29
Pediatric Surgery	1.21	95%	0.98
General Medicine	1.41	87%	0.92
Hemato-oncology	1.27	96%	0.99
Radiotherapy	1.46	96%	0.99
Emergency Medicine	1.42	93%	1.04
Pulmonary Medicine	1.48	93%	1
Dermatology	1.71	74%	0.77
Ophthalmology	1.36	81%	0.88
ENT	1.54	80%	0.86

## Discussion

Integrating knowledge of transfusion indices into clinical practice offers several benefits, including improved patient outcomes, resource utilization, and cost-effectiveness. Developing standardized protocols based on these indices can further enhance efficiency and safety in blood transfusion.

The pattern of blood and blood component usage, as well as the standards of transfusion practices, differ across various centers and countries. In present study, only the transfusion pattern of PRBC (no other blood components) was studied (as there is a policy of 100% component preparation, so no whole blood was transfused), which was contrary to other studies, such as those by Mondal et al. [14], Alcantara et al. [16], Gaur et al. [17], and Giriyan et al. [18], showing whole blood to be transfused in maximum amount.

The C/T ratio shows the number of crossmatched units transfused and should be <2.5 or as close to 1 as possible, pointing toward efficient blood utilization. This study had an overall C/T ratio of 1.53. The Department of General Surgery had the maximum C/T ratio of 2.05, and the Department of Pediatrics had the best C/T ratio of 1.2, which is similar to the study by Mondal et al. [14]. In their study, the overall C/T ratio was 1.27, with the Department of General Surgery having the maximum C/T ratio of 1.48 and the Department of Pediatrics having the most optimum C/T ratio of 1.02. The C/T ratio in the present study is within the acceptable range of C/T ratio of <2.5; however, measures to bring it as close to 1 as possible should be taken to ensure as many crossmatched PRBC units are transfused.

The present study had the maximum PRBC units utilized by the Department of Medicine and Department of Ophthalmology transfused with the minimum number of PRBC units. This is similar to the study by Alcantara et al [16], who reported the maximum utilization by the Department of Medicine and the minimum utilization by the Department of Pediatrics. In the study by Giriyan et al. [18], the Department of Medicine utilized the maximum units after the Department of Obstetrics and Gynecology, and the Department of ENT had the minimum utilization. In the study by Gaur et al. [17], the maximum blood units transfused was noted in the Department of General Surgery.

TI determines the number of blood units transfused per patient crossmatched, and TP determines the percentage of crossmatched patients transfused. A TP >30% and a TI >0.5 show efficient blood utilization. In this study, overall TP was 88% and TI was 1.15, which was 66.62% and 0.92, respectively, in the study by Bimal et al. [14]. The maximum TI was 2.47 for the Department of Gastro-surgery, while the Department of Dermatology had the minimum TI of 0.77. Alcantara et al. [16] reported a TI of 2.43.

This study had the maximum number of B-positive PRBC transfusions, followed by O-positive, A-positive, and AB-positive transfusions. Moreover, B-negative transfusions were maximum in the Rh(D)-negative group, and AB-negative transfusions were minimum. Bimal et al. [14] reported the maximum B-positive PRBC transfusions and no AB-negative transfusions.

Limitations

The largest limitation of the study was that it was a retrospective study, as some of the missing information could not be obtained from the treating clinicians. As software was not available in the department, data were collected from blood bank registries in the physical form, which was very time-consuming and incomplete on a few occasions. Specific diagnosis and specific type of surgery-related information could not be collected, as only anemia or for OT was mentioned in the diagnosis column of the blood requisition form. MSBOS could not be calculated as the specific surgery type was not mentioned. Gender-wise data could not be collected as this column was empty in many places.

## Conclusions

Quality indicators, namely, CT ratio, TP, and TI, in the present study demonstrated efficient blood utilization. Based on the transfusion indices, the departments with outlier indices can be asked to prepare specific protocols to reduce these transfusion indices. For example, in this study, the Department of General Surgery needs to send crossmatching requisitions for only those patients who are really in need and follow restrictive transfusion protocols. The Department of Gastro-surgery needs to follow restricted transfusion protocols to reduce the number of units being transfused per patient (as TI is higher). The Department of Dermatology needs to evaluate its patients for the need for blood transfusion. CMEs can be organized to educate clinicians to follow transfusion guidelines and restrictive transfusion protocols. Formulating specific guidelines for transfusion by the departments will reduce man-hours, manpower, unnecessary usage of consumable items, cost, and unnecessary transfusions. These resources can then be utilized in the specific situations where they are needed.
